# Should we offer routine hepatitis C antibody testing in men who have sex with men?

**DOI:** 10.7448/IAS.17.4.19591

**Published:** 2014-11-02

**Authors:** Christopher Ward, Vincent Lee

**Affiliations:** 1 Manchester Centre for Sexual Health, Central Manchester University Hospitals NHS Foundation Trust, Manchester, UK

## Abstract

**Introduction:**

There has been a significant rise in the number of HIV positive men who have sex with men (MSM) co-infected with hepatitis C (HCV). Most infections are thought to occur through high risk sexual practices, exacerbated by drug use. Previous data has suggested no need for routine screening in HIV negative MSM. We looked at HCV antibody testing and HCV risk assessment in all MSM clinic attenders as part of a Public Health England initiative.

**Materials and Methods:**

Routine HCV antibody testing was offered to all MSM attending a large inner city sexual health clinic from April to June 2014. Patients were asked to fill in a questionnaire assessing HCV risk. Demographic data, HIV status and STI results were collected and compared.

**Results:**

We collected 471 HCV risk assessment questionnaires during the eight-week period. The median age was 34 (range 18–71) and 403 (85.6%) were White British. Ten (2.1%) patients were known to be HCV positive, of which 3 were on treatment and 5 (1.1%) had cleared HCV. One hundred and forty-nine (31.6%) patients were HIV negative, 254 (53.9%) were HIV positive and 68 (14.5%) had unknown HIV status at time of clinic visit. In the last three months 151 (32.1%) reported unprotected receptive anal intercourse, 58 (12.3%) reported group sex, 11 (2.3%) reported receptive fisting and 32 (6.8%) reported more than 10 partners. Eighty-seven (18.5%) patients had shared notes/straws to snort drugs and 29 (6.2%) reported injecting drugs or slamming. One hundred and forty-two (30.0%) patients reported recreational drug use in the last 12 months, with cocaine, methadrone and ketamine most popular. One hundred and fifteen (24.4%) patients reported sex under the influence of recreational drugs. There were no statistical differences between HIV positive and HIV negative patients in their risk, sexual behaviour and drug use. STI screens were performed on 269 patients with nine (3.3%) new HIV diagnoses, four (1.5%) early syphilis, and 28 (10.4%) rectal gonorrhoea infections. There were three (1.1%) new HCV diagnoses, and one (33.3%) was in an HIV negative patient.

**Conclusions:**

Our results show increased risk behaviour for both HIV positive and HIV negative MSM. There are a high number of patients using party drugs, participating in group sex and not using condoms, leading to high rates of new STI diagnoses. With similar rates of risk we believe HCV testing and risk assessment should be considered in all MSM regardless of HIV status.

